# Contrasting Responses to Stress Displayed by Tobacco Overexpressing an Algal Plastid Terminal Oxidase in the Chloroplast

**DOI:** 10.3389/fpls.2020.00501

**Published:** 2020-04-28

**Authors:** Niaz Ahmad, Muhammad Omar Khan, Ejazul Islam, Zheng-Yi Wei, Lorna McAusland, Tracy Lawson, Giles N. Johnson, Peter J. Nixon

**Affiliations:** ^1^Agricultural Biotechnology Division, National Institute for Biotechnology and Genetic Engineering, Faisalabad, Pakistan; ^2^Department of Life Sciences, Sir Ernst Chain Building–Wolfson Laboratories, Imperial College London, London, United Kingdom; ^3^Soil and Environmental Biotechnology Division, National Institute for Biotechnology and Genetic Engineering, Faisalabad, Pakistan; ^4^Institute of Agricultural Biotechnology, Jilin Academy of Agricultural Science, Changchun, China; ^5^School of Life Sciences, University of Essex, Essex, United Kingdom; ^6^School of Natural Sciences, The University of Manchester, Manchester, United Kingdom

**Keywords:** PTOX, chloroplasts, chlororespiration, photoinhibition, stress tolerance

## Abstract

The plastid terminal oxidase (PTOX) – an interfacial diiron carboxylate protein found in the thylakoid membranes of chloroplasts – oxidizes plastoquinol and reduces molecular oxygen to water. It is believed to play a physiologically important role in the response of some plant species to light and salt (NaCl) stress by diverting excess electrons to oxygen thereby protecting photosystem II (PSII) from photodamage. PTOX is therefore a candidate for engineering stress tolerance in crop plants. Previously, we used chloroplast transformation technology to over express PTOX1 from the green alga *Chlamydomonas reinhardtii* in tobacco (generating line Nt-PTOX-OE). Contrary to expectation, growth of Nt-PTOX-OE plants was more sensitive to light stress. Here we have examined in detail the effects of PTOX1 on photosynthesis in Nt-PTOX-OE tobacco plants grown at two different light intensities. Under ‘low light’ (50 μmol photons m^–2^ s^–1^) conditions, Nt-PTOX-OE and WT plants showed similar photosynthetic activities. In contrast, under ‘high light’ (125 μmol photons m^–2^ s^–1^) conditions, Nt-PTOX-OE showed less PSII activity than WT while photosystem I (PSI) activity was unaffected. Nt-PTOX-OE grown under high light also failed to increase the chlorophyll *a/b* ratio and the maximum rate of CO_2_ assimilation compared to low-light grown plants, suggesting a defect in acclimation. In contrast, Nt-PTOX-OE plants showed much better germination, root length, and shoot biomass accumulation than WT when exposed to high levels of NaCl and showed better recovery and less chlorophyll bleaching after NaCl stress when grown hydroponically. Overall, our results strengthen the link between PTOX and the resistance of plants to salt stress.

## Introduction

Plastid or plastoquinol terminal oxidase (PTOX) is a non-heme diiron carboxylate protein found widely in oxygenic photosynthetic organisms that oxidizes plastoquinol (PQH_2_) to plastoquinone (PQ) and reduces molecular oxygen to water (Joët et al., 2002; Josse et al., 2003). PTOX plays an important role in chloroplast biogenesis, carotenoid biosynthesis and chlororespiration (Carol et al., 1999; Wu et al., 1999; Josse et al., 2000; Joët et al., 2002).

Upregulation of PTOX expression has been reported for several stress-tolerant plant species acclimated to harsh environments such as drought, high light and high temperature (Quiles, 2006; Ibanez et al., 2010), high salinity (Stepien and Johnson, 2009), low temperature (Streb et al., 2005; Ivanov et al., 2012), and high levels of UV light (Laureau et al., 2013). PTOX is believed to act as a stress-induced safety valve that keeps the acceptor side of PSII oxidized, thereby helping to protect PSII from photo-damage (Streb et al., 2005; Laureau et al., 2013; Johnson and Stepien, 2016; Krieger-Liszkay and Feilke, 2016). PTOX has therefore been proposed as a potential candidate for engineering stress tolerance in crop plants (Laureau et al., 2013; Johnson and Stepien, 2016; Krieger-Liszkay and Feilke, 2016).

Attempts to overexpress PTOX by nuclear transformation have been made in several systems such as Arabidopsis (Rosso et al., 2006; Stepien and Johnson, 2018) and tobacco (Joët et al., 2002; Heyno et al., 2009), but so far nuclear transformation has failed to confer stress tolerance to stress-sensitive plants. Using chloroplast transformation technology, we were able to successfully express an human influenza hemagglutinin (HA) tagged derivative of the PTOX1 encoded by the green alga *Chlamydomonas reinhardtii* (Houille-Vernes et al., 2011) in tobacco plants (Nt-PTOX-OE) and show that PTOX1 was targeted to the thylakoid membranes and was active (Ahmad et al., 2012). Somewhat surprisingly, expression of PTOX1 made plant growth susceptible to high light; an observation that was at odds with its suggested photoprotective function.

Subsequent analysis of Nt-PTOX-OE plants grown under low light suggested that PTOX1 diverts electrons from the PQH_2_ pool to oxygen thereby decreasing net forward electron flow to PSI and the rate of CO_2_ assimilation (Feilke et al., 2016). So far detailed studies on Nt-PTOX-OE have been carried out on plants grown under low light conditions or thylakoids isolated from such plants. Here we expand on these earlier studies to investigate effects on PSI and PSII function in Nt-PTOX-OE plants grown at higher light intensities that cause symptoms of chronic photoinhibition (Ahmad et al., 2012). Our results suggest that overexpression of PTOX1 in tobacco chloroplasts affects PSII but not PSI activity at higher irradiance levels. Furthermore, Nt-PTOX-OE plants are unable to increase their photosynthetic capacity when grown at higher light intensities.

Given that PTOX-based electron flux has been postulated as a mechanism to engineer salt stress tolerance in crop plants, we report here the performance of the Nt-PTOX-OE plants under NaCl stress. We have found that the Nt-PTOX-OE plants showed much higher germination rates under NaCl stress, better root length, and exhibited less chlorophyll bleaching compared to wild type. To our knowledge, this is the first report linking PTOX overexpression to salt resistance at the level of germination and root development.

## Results

### Accumulation of PTOX1 in Tobacco Leaves Grown at High Light

**FIGURE 1:**
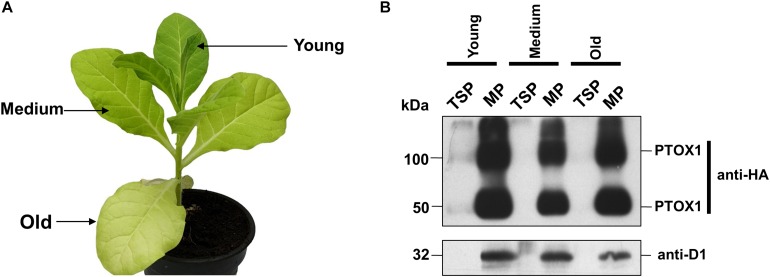
Accumulation of PTOX1 in tobacco chloroplasts under different light intensities. **(A)** Phenotype of a 10-week-old Nt-PTOX-OE plant grown at 125 μmol photons m^–2^ s^–1^. The leaves used for the isolation of soluble and membrane proteins are indicated by the arrows. **(B)** Total soluble proteins (TSP) and membrane proteins (MP) were isolated and analyzed on 12.5% denaturing acrylamide gel followed by immunoblotting. ∼10 μg of total soluble fractions while for the membrane fractions, ∼2 μg of chlorophyll was loaded per well and probed using either anti-HA (Human influenza hemagglutinin) tag (upper panel) or anti-D1 antibodies (lower panel). Dection of band in anti-HA blot at ∼100 kDa shows the possible formation of PTOX1 dimers, as reported earlier (Ahmad et al., 2012). Representative figures in each panel from one of the three biological replicates are shown here.

Nt-PTOX-OE plants expressing PTOX1 grow normally at an irradiance of 50 μmol photons m^–2^ s^–1^ (referred to here as ‘low-light’ conditions), but display stunted growth and are chlorotic, especially in older leaves, when grown at 125 μmol photons m^–2^ s^–1^ (hereafter ‘high-light’ conditions), a phenotype that can be reversed by reducing the light intensity (Ahmad et al., 2012). PTOX is an interfacial protein located on the stromal side of the non-appressed thylakoid membranes (Lennon et al., 2003). Recent work has suggested that PTOX activity might be regulated at the level of attachment of PTOX to the thylakoid membrane, promoted by increased alkalinity of the stroma induced by high light (Laureau et al., 2013; Feilke et al., 2016; Bolte et al., 2020). To examine whether the pale phenotype observed in Nt-PTOX-OE plants grown under high light (Figure 1A) could be due to the differential accumulation of PTOX1 or effects on binding of PTOX1 to the thylakoid membrane (Ahmad et al., 2012; Feilke et al., 2016), soluble and membrane proteins were extracted from Nt-PTOX-OE leaves of different ages and analyzed by immunoblotting using antibodies specific for either the thylakoid membrane-bound D1 subunit of PSII or the HA-tag which had been fused to the N-terminus of PTOX1 in Nt-PTOX-OE. The absence of detectable PTOX1 in soluble fractions and its presence in membrane fractions suggested that the PTOX1 was predominantly targeted to the membrane fraction. Furthermore, there was no evidence for drastic changes in the relative abundance of PTOX1 and D1 (on a chlorophyll basis) in the various leaves (Figure 1B).

### Nt-PTOX-OE Plants Show Decreased Levels of Photosynthetic Pigments in High Light

**FIGURE 2:**
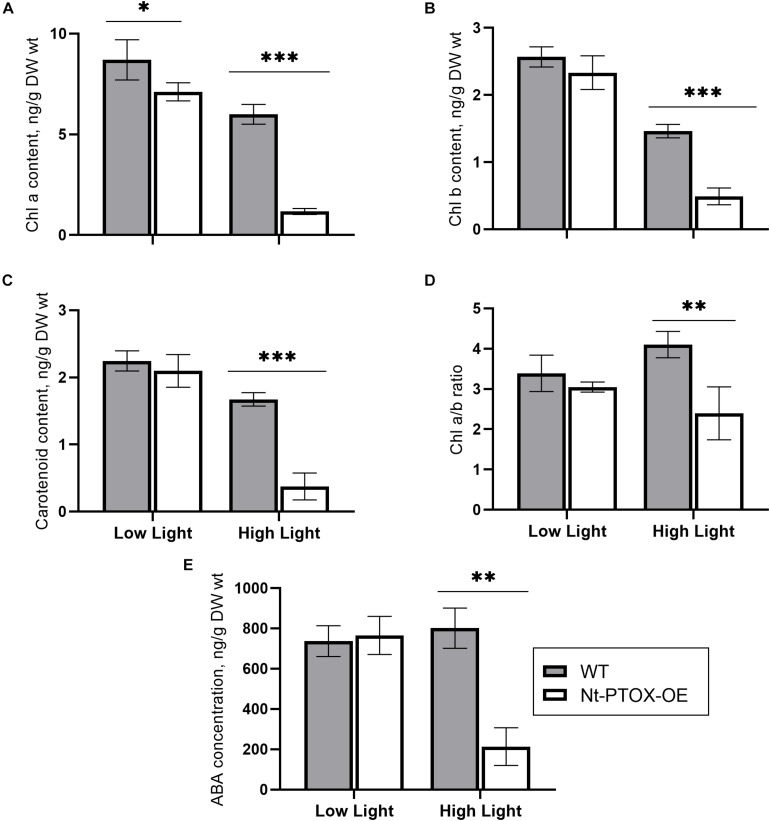
Measurements of photosynthetic pigments and ABA. WT (gray bars) and Nt-PTOX-OE plants (white bars) were grown at low light (50 μmol photons m^–2^ s^–1^) and high light (125 μmol photons m^–2^ s^–1^). Equal size leaves (3^rd^ from top) from 6 to 9 8-week-old plants were used for the extraction of pigments chlorophyll *a* **(A)**, chlorophyll *b* **(B)**, carotenoids **(C)**, chlorophyll *a/b* **(D)** and the hormone abscisic acid **(E)**. Data points represent the means ± SE of four biological replicates. ^∗^, ^∗∗^, ^∗∗∗^ significant at *P* = 0.05, 0.01 and 0.001, respectively.

PTOX activity is required for efficient phytoene desaturation and the synthesis of carotenoids (Norris et al., 1995; Kuntz, 2004). Since the leaves of tobacco plants expressing PTOX1 were visibly more yellow, we investigated whether the Nt-PTOX-OE transplastomic plants might be accumulating more carotenoids. The content of chlorophyll (Chl) and carotenoid was found to be comparable in wild type (WT) and Nt-PTOX-OE plants grown at low light intensity (Figures 2A–C; low light). However, levels of these pigments were reduced significantly in Nt-PTOX-OE plants, but not WT, when grown under high light (Figures 2A–C; high light). In addition, levels of the plant hormone, abscisic acid (ABA), which is derived from carotenoid precursors, was also significantly reduced in Nt-PTOX-OE grown under high light but not low light (Figure 2E). Overall these data suggest that the carotenoid biosynthesis pathway is down-regulated in Nt-PTOX-OE in high light.

The ratio of Chl *a/*Chl *b* was similar in both the WT and Nt-PTOX-OE plants grown under low light (Figure 2D). However, at high light, only the WT plants showed an increase in the Chl *a*/*b* ratio, which has been used as an indicator of the acclimation of the plants to high light intensities (Huner et al., 1998).

### Photosynthetic CO_2_ Assimilation in Nt-PTOX-OE Plants

**FIGURE 3:**
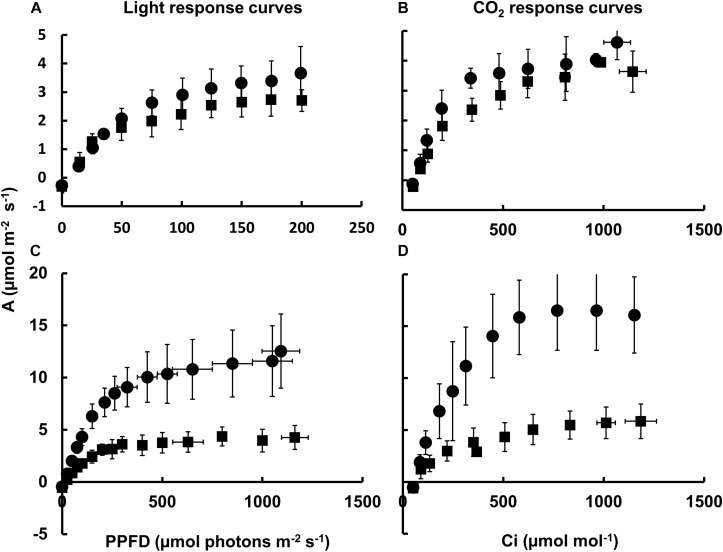
Photosynthetic response of Nt-PTOX-OE plants. Four WT (circles) and Nt-PTOX-OE transplastomic plants (squares) grown either at 50 μmol photons m^–2^ s^–1^ **(A,B)** or 125 μmol photons m^–2^ s^–1^ light intensity **(C,D)** were analyzed. Carbon assimilation rates **(A)** were determined as a function of increasing light **(A,C)** and intercellular CO_2_ concentration (*C*_i_) **(B,D)** at saturating light levels 100 μmol photons m^–2^ s^–1^ for plants grown at low light **(B)** and 1,000 μmol photons m^–2^ s^–1^ for plants grown at high light **(D)**. Data points represent the means ± SE of three biological replicates. PPFD, photosynthetic photon flux density.

In order to better understand the impact on photosynthesis in Nt-PTOX-OE plants grown under high light, we measured the rate of CO_2_ assimilation as a function of light intensity and external CO_2_ concentration using infared gas exchange. CO_2_ assimilation rates were similar in Nt-PTOX-OE and WT plants grown under low light (Figures 3A,B), although the values we obtained were generally low for tobacco (Simkin et al., 2017), possibly due to the lower light intensities used to grow the WT and Nt-PTOX-OE plants.

In contrast to WT, no increase in the light-saturated rate of photosynthesis (*A*_*sat*_) or the light and CO_2_-saturated rate of photosynthesis (*A*_*max*_) was observed in Nt-PTOX-OE grown under high light intensities compared to plants grown at low light (Figures 3C,D), suggesting a defect in acclimation to high light growth conditions, in line with the observed lack of increase in the Chl *a/b* ratio at higher irradiances (Figure 2D).

### Photosynthetic Efficiency Does Not Increase in Nt-PTOX-OE Plants Under High Light

**FIGURE 4:**
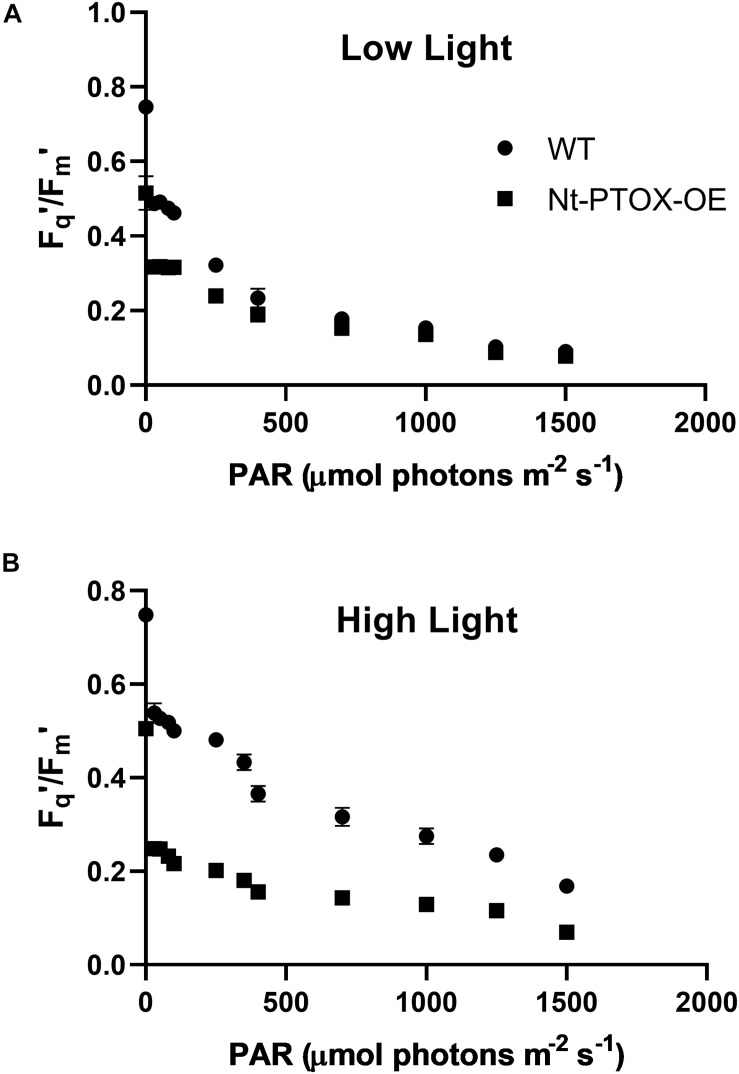
Determining photosynthetic efficiency of Nt-PTOX-OE plants. Four WT (circles) and Nt-PTOX-OE plants (squares) were grown either at 50 μmol photons m^–2^ s^–1^ **(A)** or 125 μmol photons m^–2^ s^–1^ **(B)** light intensity. Data points represent the means ± SE of three biological replicates.

Possible effects on acclimation to high light were also investigated by measuring the photosynthetic efficiency in Nt-PTOX-OE plants grown at high and low light by determining the PSII operating efficiency (*F_q′_*/*F_m’_*) at steady state across a range of irradiance levels (Figure 4). At a given photosynthetic photon flux density (PPFD), *F_q′_*/*F_m’_* is a good estimate of the relative linear electron flux through PSII (Baker, 2008). *F_q′_*/*F_m’_* values in Nt-PTOX-OE were comparable to WT plants when plants were grown under low light (Figure 4A). In contrast, WT plants grown at high irradiance consistently displayed a higher value for *F_q′_*/*F_m’_* at any given irradiance than the equivalent Nt-PTOX-OE plants (Figure 4B).

Taken together, we conclude that Nt-PTOX-OE plants are unable to adjust their photosynthetic apparatus correctly to the growth light they experience.

### Effect of PTOX1 Overexpression on Photosystem I and II Photochemistry

**FIGURE 5:**
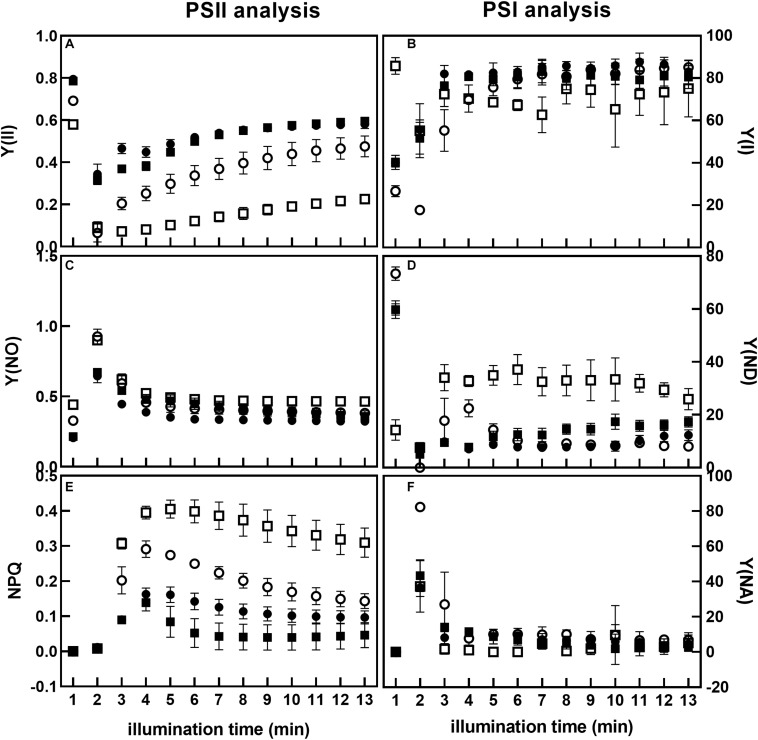
Analysis of PSII and PSI photochemistry of Nt-PTOX-OE plants. Nine WT (circles) and Nt-PTOX-OE (squares) plants were grown either at 50 μmol photons m^–2^ s^–1^ (closed symbols) or 125 μmol photons m^–2^ s^–1^ (open symbols) light intensity. Plants were dark-adapted for 30 min before the fluorescence measurements were carried out. Measurements in plants grown at low light were made at 26 μmol photons m^–2^ s^–1^ while in high light grown plants, measurements were conducted at 65 μmol photons m^–2^ s^–1^. Data points represent the means ± SE of three independent biological replicates. Y(II), quantum yield of PSII **(A)**; Y(I), quantum yield of PSI **(B)**; Y(NO), non-regulated heat dissipation **(C)**; Y(ND), donor side limitation **(D)**; Y(NPQ), non-photochemical quenching **(E)**; Y(NA), acceptor side limitation **(F)**.

We then studied PSII and PSI photochemistry in more detail by measuring the quantum yield of PSII [Y(II)], non-regulated heat dissipation [Y(NO)], and non-photochemical quenching [Y(NPQ)] at a sub-saturating light intensity (Figure 5). PSI photochemistry was studied by measuring the quantum yield of PSI Y(I), acceptor side limitation Y(NA), and donor side limitation Y(ND) of plants grown under low and high irradiance.

During induction at a sub-saturating irradiance, PSII quantum yield Y(II) in Nt-PTOX-OE plants grown under low light was similar to WT plants (Figure 5A). For both WT and Nt-PTOX-OE plants grown at high light, Y(II) was lower than plants grown at low light, but the reduction in Y(II) was more pronounced in Nt-PTOX-OE plants. In contrast, PSI quantum yield Y(I) remained largely unchanged in Nt-PTOX-OE and WT plants grown under low light as well as high light (Figure 5B). Non-regulated heat dissipation, Y(NO), showed no difference between WT and Nt-PTOX-OE plants grown in either light condition (Figure 5C). Examination of NPQ showed that NPQ was marginally lower in Nt-PTOX-OE than WT under low light (Figure 5E). However, NPQ was much higher in Nt-PTOX-OE plants when grown under high light compared to WT. The donor side limitation Y(ND) and acceptor side limitation Y(NA) parameters of PSI showed that Y(ND) remain unchanged in both the Nt-PTOX-OE and WT under low light, but increased significantly in Nt-PTOX-OE plants grown under high light (Figure 5D). The accepter side limitation at PSI remains unchanged under both light conditions (Figure 5F). These observations clearly show that the reduction in the growth in Nt-PTOX-OE plants under high light is mainly due to the suppression of the PSII activity rather than PSI.

### PTOX1 Confers Salt Tolerance in Seedlings and Mature Plants

PTOX has been viewed as an important candidate for engineering stress tolerance in crop plants (Johnson and Stepien, 2016; Krieger-Liszkay and Feilke, 2016). However, attempts to transfer PTOX-mediated stress tolerance into stress sensitive species such as Arabidopsis (Rosso et al., 2006; Stepien and Johnson, 2018) and tobacco (Joët et al., 2002; Heyno et al., 2009) have so far not been successful. To test whether Nt-PTOX-OE plants might show increased stress tolerance, we challenged Nt-PTOX-OE plants with different NaCl concentrations; both at the stage of germination as well as whole plants grown hydroponically.

**FIGURE 6:**
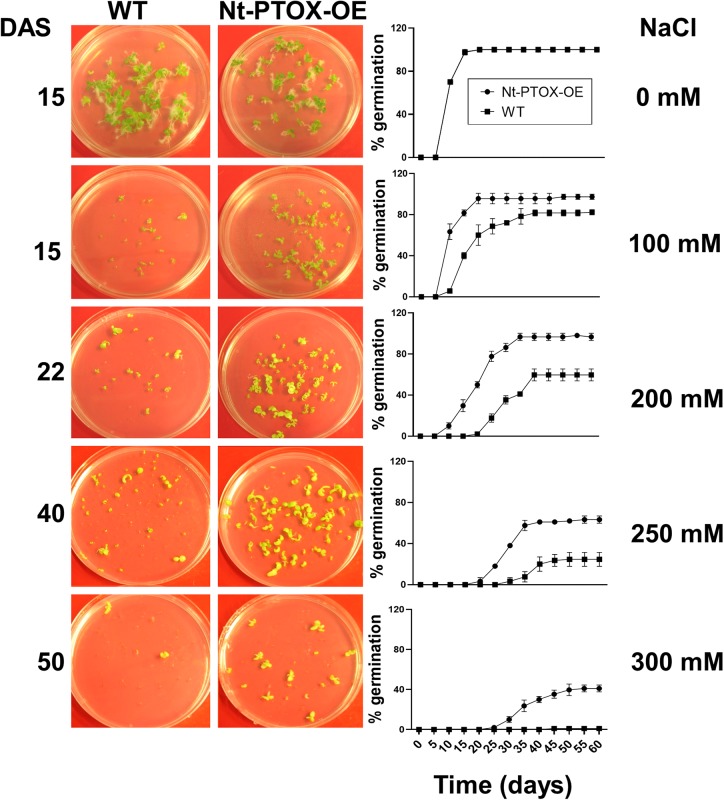
Effect of NaCl on germination of Nt-PTOX-OE. Seeds of Nt-PTOX-OE and WT plants were germinated on MS supplemented with 0, 100, 200, 250, and 350 mM NaCl. Plates were placed at room temperature in a growth room at 50 μmol photons m^–2^ s^–1^ light intensity. Pictures were taken at different days indicated against each salt concentration. Germination of WT (squares) and Nt-PTOX-OE (circles) is shown in percentage from three independent experiments. Data points represent the means ± SE of three independent biological replicates. DAS, days after sowing.

Seeds of Nt-PTOX-OE and WT plants were germinated on Murashige and Skoog (MS) solid medium supplemented with 0, 100, 200, 250, 300, 400, and 500 mM NaCl. Germination was completed within 2 weeks using control plates lacking added NaCl, but was significantly delayed in plates where NaCl was added and did not occur at all beyond 300 mM NaCl. Nt-PTOX-OE showed higher germination rates compared to WT at all levels of additional NaCl (Figure 6) with the effect more pronounced at higher concentrations.

**FIGURE 7:**
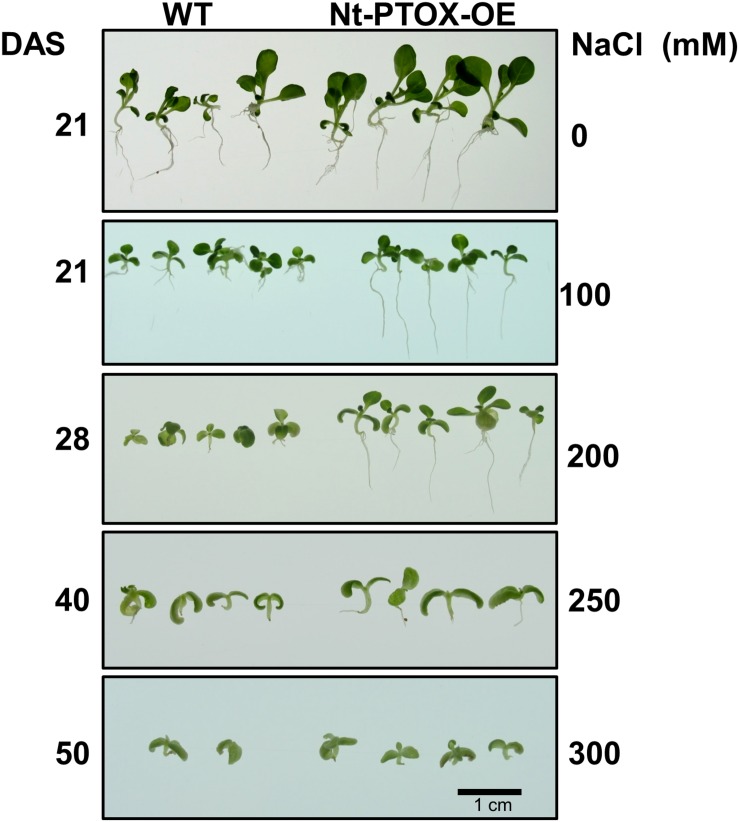
Effect of NaCl on root and shoot length of Nt-PTOX-OE. Seeds of Nt-PTOX-OE and WT plants were germinated on MS supplemented with 0, 100, 200, 250, and 350 mM NaCl at 25°C temperature and 50 μmol photons m^–2^ s^–1^ light intensity. The labeling on the left-hand side shows the number of days after sowing while on the right shows NaCl concentration used. The experiment was repeated three times. DAS, days after sowing.

The effect of NaCl concentration on germination was almost completely mirrored in seedling growth. Although root length was reduced in both Nt-PTOX-OE and WT seedlings at higher salt concentrations (Figure 7), Nt-PTOX-OE seedlings were less affected than WT seedlings especially at higher NaCl concentrations.

**FIGURE 8:**
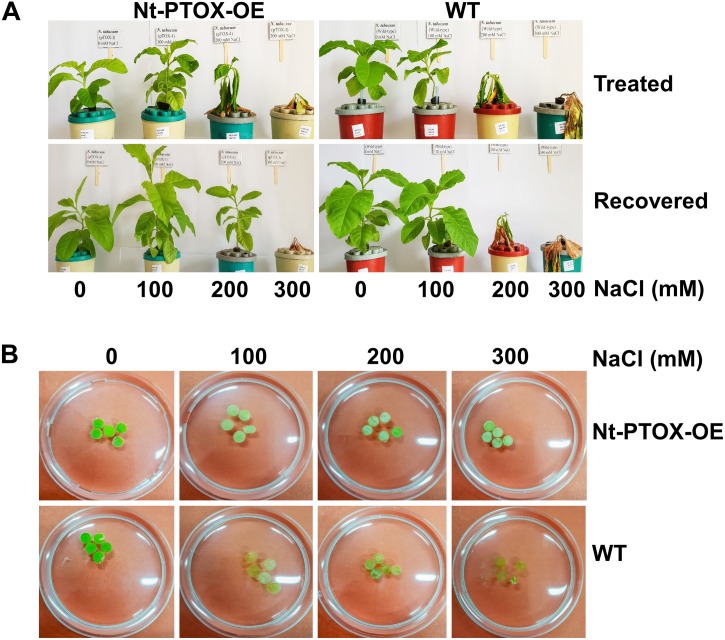
Tolerance to NaCl stress in hydroponics and leaf disk assays. Four-week-old Nt-PTOX-OE and WT plants raised under 50 μmol photons m^–2^ s^–1^ were transferred to hydroponic solution **(A)** supplemented with indicated NaCl concentrations. Plants were allowed to recover for 1 week by growing on NaCl-free medium. **(B)** The leaf disks were prepared from the plants when they were established in the hydroponics (after 1 week) before imposing NaCl stress. Only one representative figure in each panel from three independent biological replicates is shown here.

The effect of NaCl was also studied in mature plants. Six-week-old plants were established in hydroponic solutions for 1 week. After 1 week, the plants were exposed to 0, 100, 200, 300 mM NaCl for a period of 1 week. The NaCl toxicity symptoms were milder in Nt-PTOX-OE plants at 200 mM NaCl compared to WT (Figure 8A). After 1 week, the salts were removed, and plants allowed to recover in a salt-free solution. During the recovery phase, the Nt-PTOX-OE plants showed much better recovery compared to WT plants particularly at 200 mM NaCl.

Bleaching of chlorophyll in leaf disks obtained from plants grown hydroponically under low light and exposed to increasing levels of NaCl was also reduced in Nt-PTOX-OE compared to WT plants (Figure 8B). Overall these data suggest a role for PTOX1 in protecting Nt-PTOX-OE plants against salt stress.

## Discussion

PTOX has emerged as a potential candidate to confer tolerance against abiotic stresses such as high light and high salt. However, attempts to engineer PTOX-mediated stress-tolerance in stress-sensitive plants has so far not been successful (Joët et al., 2002; Rosso et al., 2006; Heyno et al., 2009; Stepien and Johnson, 2018). In fact overexpression of PTOX1 in tobacco, in mutant Nt-PTOX-OE, renders plant growth and PSII activity [as determined by maximum quantum efficiency of PSII (*F*_*v*_/*F*_*m*_)] more sensitive to high light (Ahmad et al., 2012). Subsequent work on these plants by Feilke et al. (2016) showed that PTOX1 was highly active in these plants and competed efficiently with forward electron flow to PSI. Although the activities of PSII and PSI remain unaffected in thylakoids isolated from low-light grown plants (Feilke et al., 2016), potential effects on PSI at high light have not been studied. Here we have shown that PSII activity is much more sensitive to light stress (Figure 4) than PSI activity (Figure 5) in Nt-PTOX-OE. Based on this and previous work (Ahmad et al., 2012), we think this is likely to reflect photoinhibition, though differences in PSII accumulation are liable to affect this. The increase in donor side limitation of PSI, Y(ND), observed in Nt-PTOX-OE at high light suggests either the induction of a regulatory mechanism to control electron flow toward PSI (Joliot and Johnson, 2011) or the suppression of PSII activity, possibly due to impacts on the repair of damaged PSII by reactive oxygen species (ROS) produced by PTOX. This might be one reason why the PTOX level is low under natural/unstressed conditions (∼1 PTOX protein molecule per 100 PSII molecules) (Lennon et al., 2003; Krieger-Liszkay and Feilke, 2016).

One striking phenotype of the Nt-PTOX-OE plants we observed was a perturbed ability for photosynthesis to acclimate to high light growth (Figure 4B) as indicated by lack of increase in the Chl *a/b* ratio (Figure 2) and CO_2_ assimilation rates (Figure 3). Further work is needed to understand the molecular basis of this effect but one reason for this could be that changes in redox state of the PQ pool by PTOX1 (Feilke et al., 2016) might have effects on nuclear gene expression (Pfannschmidt et al., 1999; Frigerio et al., 2007) or, as shown recently, on the synthesis of chlorophyll (Brzezowski et al., 2019). However other effects cannot yet be excluded including the possibility that PTOX1 from *Chlamydomonas* could be catalytically different from PTOX found in higher plants or over-expression might have pleiotropic effects in the chloroplast such as aberrant binding to the Cyt *b*_6_*f* complex or perturbation of normal gene expression in the chloroplast due to the competing expression of PTOX1 which is driven by a strong chloroplast promoter (Ahmad et al., 2012).

A further dramatic phenotype displayed by the Nt-PTOX-OE plants was enhanced resistance to NaCl stress at the levels of germination (Figure 6), root and shoot lengths (Figure 7), recovery from NaCl treatment (Figure 8A) and chlorophyll bleaching (Figure 8B). This is the first time that expression of PTOX in a plant has been shown to confer salt tolerance in a salt-sensitive plant. Seed germination is a crucial stage in the life of a plant, and is more sensitive to stresses compared to other stages of plant development (Ibrahim, 2016). The apparent salt tolerance could be due to indirect effects of expressing PTOX1, possibly in non-photosynthetic plastids or via changes in hormone accumulation. PTOX is crucial in the early stages of plant development where newly synthesized chlorophyll needs protection from photo-oxidation (Carol and Kuntz, 2001; Kuntz, 2004; Rosso et al., 2009). In non-photosynthetic tissues or at early stages of plant development where photosynthetic electron transport (PET) is not fully active, PTOX has been shown to be a major co-factor for phytoene desaturases (PDS) and ζ carotene desaturase (ZDS) involved in carotenoid desaturation reactions (Carol and Kuntz, 2001). Therefore, the overexpression of PTOX1 in seedlings might be driving the NaCl resistant phenotype. The decrease in pigment content as well as ABA levels (Figure 2) in adult plants suggests PTOX1 was unable to enhance carotenoid biosynthesis, possibly because of upstream effects on acclimation.

Stepien and Johnson (2018) have recently shown that heterologous over-expression of PTOX in plants in itself does not provide salt tolerance. The ability to confer salt tolerance in naturally salt tolerant species, *Eutrema salsugineum*, appears to be dependent on the activation and relocation of *E. salsugineum* PTOX polypeptide to the grana where it can interact more closely with PSII and divert electrons to oxygen. Knowledge of the factors involved in regulating this process will be an important step in improving the effectiveness of PTOX-mediated tolerance against environmental stresses.

In summary, we have observed contrasting responses to stress when *C. reinhardtii* PTOX1 was overexpressed in tobacco chloroplasts. Plant growth is more sensitive to high light but is more resistant to salt stress.

## Materials and Methods

### Plant Material and Growth Conditions

Homoplasmic Nt-PTOX-OE plants of *Nicotiana tabacum* cv. Petit Havana expressing PTOX1 in their chloroplasts generated previously (Ahmad et al., 2012) were grown in sterile Murashige and Skoog (MS) media containing 8 g L^–1^ agar, 30 g L^–1^ sucrose, and 500 mg L^–1^ spectinomycin under controlled environment conditions as reported earlier (Ahmad et al., 2012). Untransformed wildtype plants, WT, were used as controls. The WT and Nt-PTOX-OE seeds were sterilized before sowing. For low light, the seeds were germinated in a growth room at 25°C, 16 h light/8 h dark, a photosynthetic photon flux of 50 μmol photons m^–2^ s^–1^ and at 30% humidity. For high light, plants were grown in a greenhouse in plastic pots filled with Levington F2+S Seed and Modular Compost pH 5.3–5.7 (Scotts, United Kingdom) supplemented with medium sized Vermiculite pH 6.0 (2–5 mm, density 100 kg m^–3^) (Sinclair, United Kingdom) at a ratio of 4:1 at 25/20°C (day/night) in a 16 h photoperiod at a photosynthetic photon flux of 125 μmol photons m^–2^ s^–1^, and 40% humidity. For germination in compost, seeds were mixed with sand and sprinkled on pots filled with compost followed by irrigation. Plants were irrigated regularly and positions were rotated daily.

### Protein Extraction, SDS-PAGE, and Western Blot Analysis

Leaf disks from two fresh and fully expanded leaves of three independently grown Nt-PTOX-OE plants were ground into fine power in liquid nitrogen. Total soluble proteins (TSP) and membrane proteins (MP) were extracted and the protein concentration was determined as described earlier (Ahmad et al., 2012). Proteins were loaded on an equal protein concentration basis for TSP and equal chlorophyll content basis for the MPs, separated by 12.5% (w/v) SDS-PAGE, transferred to a nitrocellulose membrane using an iBlot^®^ Dry Blotting System (Invitrogen, United States) and probed using either anti-HA (Human influenza hemagglutinin) tag antibodies or D1 antibodies (Roche Applied Science, Germany). Broad-range pre-stained multicolor molecular weight standards, Spectra^TM^ (Fermentas, United States), were run alongside samples to determine the sizes of protein bands. The gels were stained with Coomassie Brilliant Blue ‘R-250.’ Proteins were immuno-detected using horseradish peroxidase (HRP) conjugated to secondary antibodies raised against rabbit IgG and an enhanced chemiluminescence (ECL) detection kit (Amersham Pharmacia, United Kingdom) with the signal captured by X-ray film (Kodak, United States).

### Chlorophyll Fluorescence Measurements

Chlorophyll fluorescence measurements were performed on 4–6 week-old Nt-PTOX-OE and WT plants using a pulse-modulated fluorometer (DUAL PAM 2000, Walz, Germany) equipped with a DUAL-PAM-100 measuring system. All measurements were carried out at room temperature and repeated using at least three biological replicates of three plants per replicate. The plants were dark-adapted for at least 30 min before the measurements were carried out.

### Determination of Photosynthetic Capacity

Photosynthesis was measured as a function of increasing PPFD (*A/Q* response cruve) using the 6400 TX portable gas exchange system (Li-Cor). Leaf temperature (25 ± 1°C) and CO_2_ concentration (400 μmol mol^–1^) were maintained by a gas analyzer. Leaves were first stabilized at 1,000 μmol photons m^–2^ s^–1^ irradiance, after which CO_2_ assimilation rate (*A*) was measured at the following 12 PPFD levels: 0, 30, 50, 80, 100, 250, 350, 400, 700, 1,000, 1,250, and 1,500 μmol photons m^–2^ s^–1^. Measurements were recorded after *A* had reached to a new steady-state level (approximately 1–3 min) before changing to a new PPFD level. The experiment was carried out on four independently grown plants.

The response of *A* to *C*_*i*_ was measured at 1,000 μmol photons m^–2^ s^–1^ light intensity (CIRAS-1; PP Systems). Measurements of *A* were made first by stabilizing leaves to achieve a stable signal at ambient CO_2_ concentration of 400 μmol mol^–1^, before CO_2_ was decreased in a stepwise manner to 300, 200, 150, 100, and 50 μmol mol^–1^ before returning to the initial value and then increased to 500, 600, 700, 800, 900, 1,000, 1,100, and 1,200 μmol mol^–1^. CO_2_ concentration and leaf temperature (25 ± 1 °C) were maintained by the gas analyser. Measurements were recorded after *A* had reached to a new steady-state level (appox. 1–3 min) before changing to the next CO_2_ level. The experiment was carried out on three independent biological replicates.

### Pigment Extraction and Quantification

The extraction and measurements of photosynthetic pigements such as Chl *a*, Chl *b*, and total carotenoids (X + C = xanthophylls and carotenes) from WT and Nt-PTOX-OE transplastomic plants were carried out spectrophotometrically from two leaves of each plant in four biological replicates as described by Lichtenthaler and Buschmann (2001).

### ABA Measurements

The ABA measurements were carried out following the method described by Forcat et al. (2008). Briefly, leaves were harvested into liquid nitrogen and freeze dried immediately. Samples were ground into fine powder in a bead beater (TissueLyser II, Qiagen) with 3 mm tungsten beads at 25 Hz/s for 3 min. A 10-mg sample was obtained in 1.5 ml microfuge tube and was extracted with 400 μl of 10% methanol containing 1% acetic acid with 1 ng of ^2^H_6_ ABA, 10 ng of ^2^H_2_ jasmonic acid (JA) and 13.8-ng ^2^H_4_ salicylic acid (SA). Each treatment also included an extraction control with no plant material. Samples were extracted in the bead beater for 2 min at 25 Hz/s, incubated on ice for 30 min and then centrifuged at 13,000 *g* for 10 min at 4°C. The supernatant was removed and the pellet was extracted again. Both extractions after pooling supernatants give 90–95% recovery of the analytes.

A 50-μl sample was drawn and analyzed by HPLC-electrospray ionization/MS-MS using an Agilent 1100 HPLC (Agilent Technologies, United States), which was attached to an Applied Biosystems Q-TRAP^TM^ 2000 (Applied Biosystems, United States). Chromatographic separation was carried out using Luna^®^ 3 μm C18(2) 100 Å LC Column 100 mm × 2.0 mm (Phenomenex, United States) at 35°C. The solvent gradient used was 100%A (94.9% H_2_O: 5% CH_3_CN: 0.1% CHOOH) to 100%B (5% H_2_O: 94.9% CH_3_CN: 0.1% CHOOH) over 20 min. Solvent B was held at 100% for 5 min then the solvent returned to 100% A for 10 min equilibration prior to the next injection. The solvent flow rate was 200 μl/min. To reduce contamination of the MS, the first 2 min of the run was directed to waste using the inbuilt Valco valve.

Analysis of the compounds was based on appropriate Multiple Reaction Monitoring (MRM) of ion pairs for labeled and endogenous JA, SA and ABA using the following mass transitions; ^2^H_2_-JA 211 > 61, JA 209 > 59, ^2^H_4_ SA 141 > 97, SA 137 > 93, ^2^H_6_ ABA 269 > 159, ABA 263 > 153, SA-glyc 299 > 93. The MS was operated in the negative mode using Turbo-Ionspray^TM^ as the ion source. Optimal conditions were determined using the Quantitative Optimization feature of the Analyst software both by infusing standards into the MS by syringe pump and injecting standards into a 200-μl/min flow of 50% Solvent A and 50% Solvent B.

The optimized conditions were as follows: temperature 400°C, Ion source gas 1 50 psi, Ion source gas 2 60 psi, Ion spray voltage −4500 V, curtain gas 40 psi, CAD gas setting 2; the DP (−25 V), EP (−9), and CEP (−2) were held constant for all transitions. Collision energies (CE) and dwell times (DT) were specific for each compound/internal standard pair, the parameters used were JA CE-25, DT 100 ms, ABA CE-17, DT 250 ms and SA CE-38, DT 50 ms. Data were acquired and analyzed using Analyst 1.4.2 software (Applied Biosystems, United States).

### Salt Tolerance Assays

Nt-PTOX-OE and WT seeds were germinated under 50 μmol photons m^–2^ s^–1^ in MS medium containing 0, 100, 200, 300, 400, and 500 mM NaCl under controlled environment conditions in three replicates. The positions of the plates were rotated daily. For salt tolerance in hydroponics, seeds were grown on compost in a green house under 50 μmol photons m^–2^ s^–1^. 2–3 week old plants were transferred to hydroponic culture containing 1/10th Hoagland solution. When plants were successfully established in hydroponics, 24 plants of similar size were selected, divided into three groups and each group was exposed to 0, 100, 200, and 300 mM NaCl solution for 15 days. Old medium was replaced with the fresh medium on regular basis. After that plants were allowed to recover on salt free medium for 1 week. For the leaf disk assay, five feaf disks (approximately 1 cm) were obtained from equal size fully expanded fresh leaves (4-week-old plants) from three idependent plants grown in hydroponics before initiating the NaCl stress, and spread into three replicates. The disks were floated in 10 ml distilled water with or without NaCl at the following concentrations: 0, 100, 200, 300 mM for 72 h under continuous light at 25 ± 1°C. The position of the plates was rotated daily.

### Statistical Analysis

All statistical analyses were performed using two-way ANOVA at α = 0.05 using statistical software SPSS v18.

## Data Availability Statement

All datasets generated for this study are included in the article/supplementary material.

## Author Contributions

All authors listed have made a substantial, direct and intellectual contribution to the work, and approved it for publication.

## Conflict of Interest

The authors declare that the research was conducted in the absence of any commercial or financial relationships that could be construed as a potential conflict of interest.
